# Supplementary material for: Association between Serum 6:2 Chlorinated Polyfluorinated Ether Sulfonate Concentrations and Lung Cancer
Source: Toxics. 2024 Aug 19;12(8):603. doi: 10.3390/toxics12080603 (PMC11359344; doi:10.3390/toxics12080603)
Supplement: Supplementary file 1 [file toxics-12-00603-s001.zip › toxics-3123518-supplementary.pdf]

SUPPORTING INFORMATION

Association between Serum 6:2 Chlorinated Polyfluorinated Ether Sulfonate Concentrations and Lung Cancer

Weili Mao 1, Jianli Qu 2, Ruyue Guo 2, Yuanchen Chen 2, Hangbiao Jin 2 and Jingyan Xu 1,*

1 Department of Pharmacy, Quzhou Affiliated Hospital of Wenzhou Medical University, Quzhou People's Hospital, Quzhou 324000, China

2 Key Laboratory of Microbial Technology for Industrial Pollution Control of Zhejiang Province, College of Environment, Zhejiang University of Technology, Hangzhou 310032, China

Standards and reagents. 6:2 chlorinated polyfluorinated ether sulfonates (6:2 Cl-PFESA) and the corresponding mass-labeled internal standards were purchased from Wellington Laboratories (Guelph, ON, Canada). Acetonitrile, Methanol (HPLC-grade), formic acid, and ammonium acetate were obtained from J&K Scientific Co. Ltd. (Shanghai, China) and ANPEL Laboratory Technologies Inc. (Shanghai, China). Milli-Q water (18 MΩ) was used in the whole experiment. Standard reference material (SRM1957, organic contaminants in non-fortified human serum) was purchased from the National Institute of Standards and Technology (NIST, USA).

Sample pre-treatment. Prior to extraction, 200 μL of serum sample was spiked with internal standards (1.5 ng each) and then shaken by hand until completely mixed. After that, 4 mL of acetonitrile was added to the serum samples. The mixture was vortexed, sonicated (53 kHz) for 30min, and centrifuged at 4000 r/min for 10min. The supernatant was transferred to a new 10 mL PP tube (Biosharp, Beijing, China). The above extraction step was repeated once again with 4 mL of methanol. The eluent was evaporated to near-dryness using gentle nitrogen. The residue was reconstituted in 50 μL of methanol for instrumental analysis.

Instrumental analysis. Quantification of 6:2 Cl-PFESA was performed on an ultraperformance liquid chromatography system (ACQUITY, UPLC) coupled to a tandem mass spectrometer (XEVO_TQS, MS/MS; Waters Co., Milford, MA, USA). A 10 μL aliquot of the sample extract was injected into an Ascentis Express F5 PFP column (2.7 μm, 90 Å, 10 cm × 2.1 mm; Sigma-Aldrich, Canada) for chromatographic separation, and the temperature of the column was maintained at 40 °C. The mobile phase consisted of water (containing 0.1% formic acid; A) and methanol (B). The elution gradient started at 20% B, then ramped up to 40% B by 1.0 min, increased to 85% B by 11 min, increased to 100% B by 12 min, and was held at 100% B for 2 min; finally, it was returned to the initial condition. The flow rate of the mobile phase was 0.3 mL/min. The mass spectrometer was operated in the electrospray ionization (negative) and multiple reaction monitoring modes.

Table S1. The Precursor and Product Ions of Analytes for Instrumental Analysis. Quantifying Ions are Highlighted in Bold.

Compound	Precursor ion	Product ion	Cone energy (V)	Collision energy (eV)
6:2 Cl-PFESA	531	**351**, 83	12	24

Table S2. Limits of Detection (LOD), Spiked Concentrations and Recoveries of 6:2 Cl-PFESA in Human serum (*n* = 5).

	Spiked concentration (ng/mL)	Recovery (%) of human urine (*n* = 5)		LOD (ng/mL)
		Mean ± SD	Range	
6:2 Cl-PFESA	1.0, 10	104 ± 10.1	95–113	0.021

Table S3. Precision of Analytical Methods for 6:2 Cl-PFESAs Spiked at Around LOD. RSD Means Relative Standard Deviation.

	Accuracy (%) [a]	Precision (RSD; %)	Intra-day (RSD; %) [b]	Inter-day (RSD; %) [b]
**6:2 Cl-PFESA**	107	8.4	2.4-9.8	5.8-13

[a] mean value of quintuplicate analysis. [b] Intra-day (24-hour) precision: n = 5. Inter-day (3 weeks) precision: n = 5.

Table S4. Distributions of serum 6:2 Cl-PFESA concentrations among the cases and controls (n = 906).

Characteristics	Control (n = 604)	Case (n = 302)	Total (n = 906)
**Detection rate [n (%)]**	597 (98.7)	305 (98.7)	902 (98.7)
**Minimum**	< LOD	< LOD	< LOD
**Percentiles**			
5th	0.21	0.25	0.22
25th	1.3	1.5	1.4
50th	2.9	3.2	3.0
75th	6.0	6.2	6.1
95th	12	12	12
**Maximum**	26	25	26

Table S5. Association between serum concentrations of 6:2 Cl-PFESA and lung cancer risk stratified by alcohol consumption habit.

	Cases/controls	Crude	Adjusted
	(*n*)	OR (95% CI)	OR (95% CI)
Alcohol consumption habit			
**Drinker**			
Q1 (< 1.3)	14/46	reference	reference
Q2 (1.3–2.9)	16/47	1.11 (0.49, 2.55)	1.20 (0.48, 3.00)
Q3 (2.9–6.0)	26/47	1.81 (0.84, 3.91)	1.83 (0.78, 4.29)
Q4 (> 6.0)	27/46	1.92 (0.89, 4.14)	2.20 (0.94, 5.16)
p for trend		0.070	0.049
**Nondrinker**			
Q1 (< 1.3)	42/104	reference	reference
Q2 (1.3–2.9)	52/105	1.22 (0.75, 1.99)	1.16 (0.70, 1.93)
Q3 (2.9–5.9)	62/105	1.46 (0.90, 2.35)	1.40 (0.85, 2.30)
Q4 (> 5.9)	63/104	1.50 (0.93, 2.41)	1.46 (0.90, 2.38)
***p* for trend**		0.109	0.123
***p* for interaction**		0.913	0.957

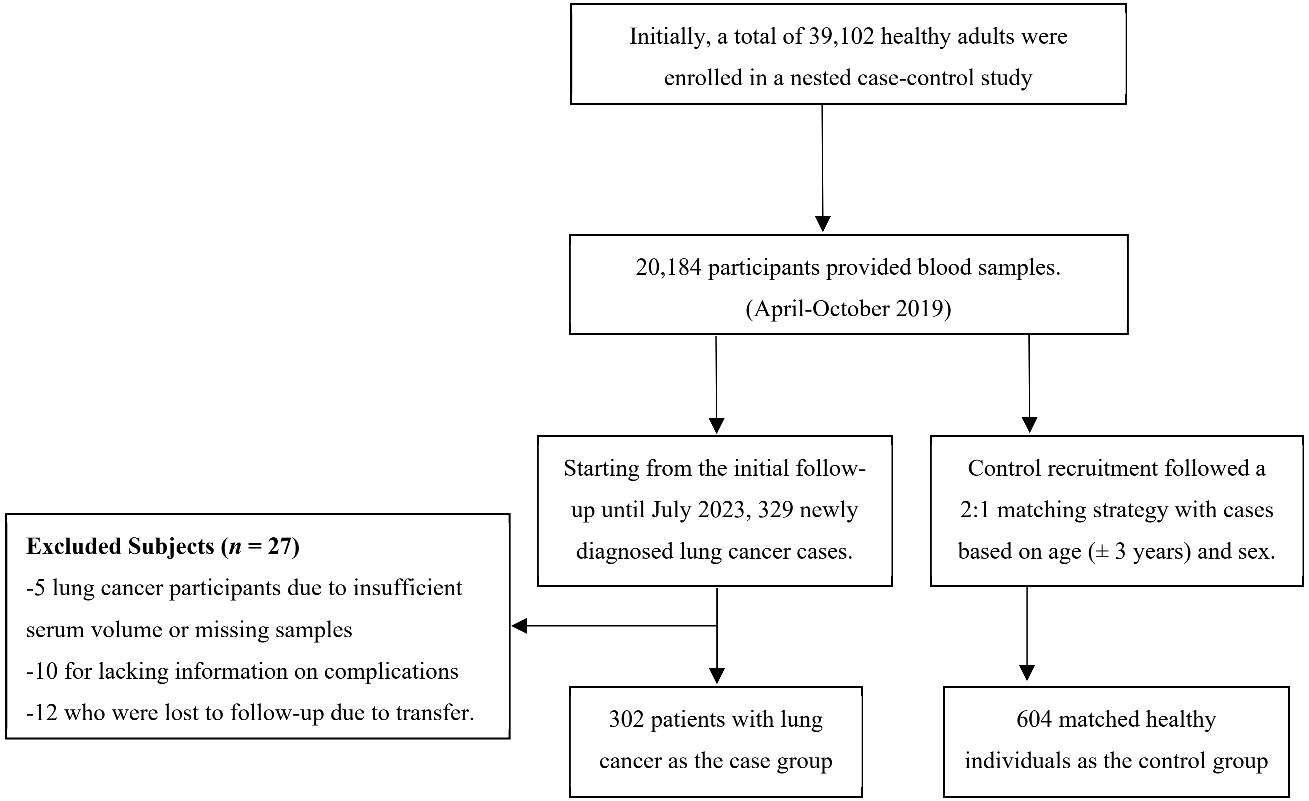

Figure S1. Flow chart of the inclusion-exclusion process of the case and control group.

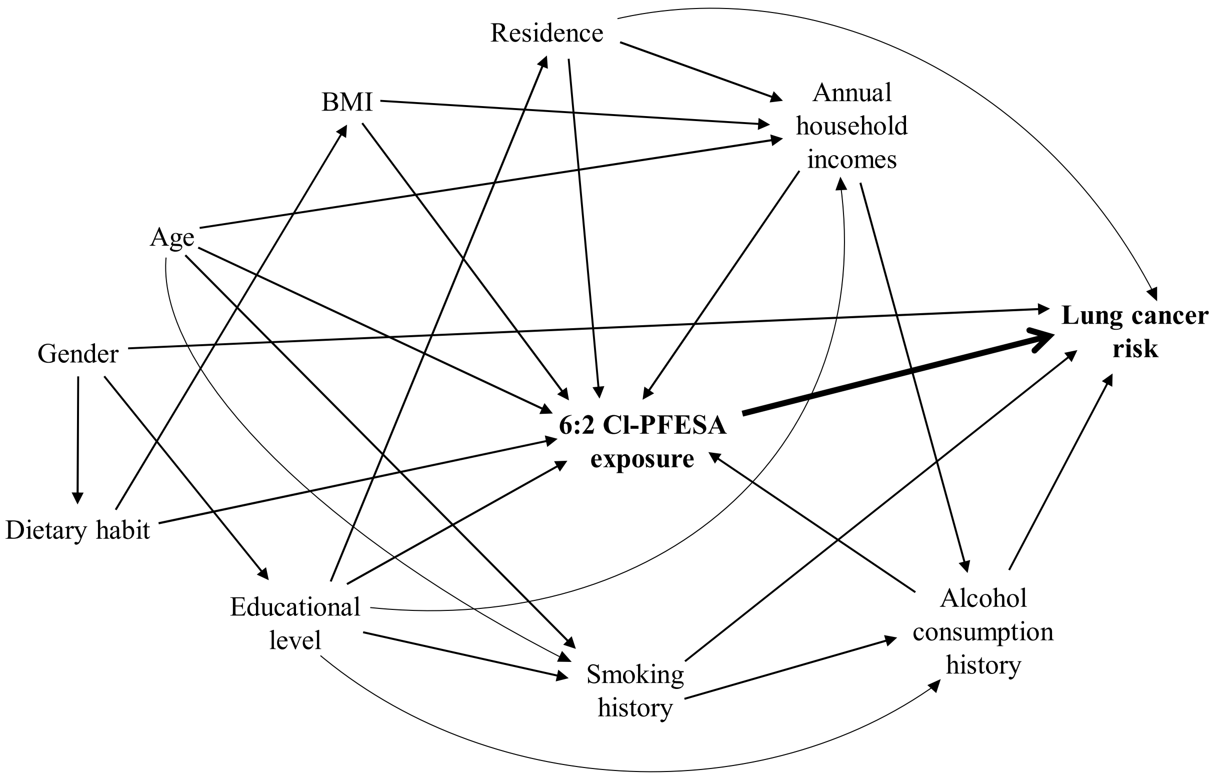

Figure S2. The directed acyclic graph (DAG) for potential confounders considered in the statistical analyses.
